# Effects of Process Cutting Parameters on the Ti-6Al-4V Turning with Monolithic Driven Rotary Tool

**DOI:** 10.3390/ma15155181

**Published:** 2022-07-26

**Authors:** Richard Joch, Michal Šajgalík, Andrej Czán, Jozef Holubják, Miroslav Cedzo, Robert Čep

**Affiliations:** 1 Department of Machining and Manufacturing Technology, Faculty of Mechanical Engineering, University of Žilina, Univerzitná 1, 010 26 Žilina, Slovakia; michal.sajgalik@fstroj.uniza.sk (M.Š.); andrej.czan@fstroj.uniza.sk (A.C.); jozef.holubjak@fstroj.uniza.sk (J.H.); miroslav.cedzo@fstroj.uniza.sk (M.C.); 2 Faculty of Mechanical Engineering, VŠB—Technical University of Ostrava, 70800 Ostrava, Czech Republic; robert.cep@vsb.cz

**Keywords:** titanium alloy, rotary tool, turning, actively driven tool

## Abstract

Machining with rotating tools appears to be an efficient method that employs a non-standard kinematic turning scheme. It is used in the machining of materials that we classify in the category of difficult to machine. The titanium alloy Ti-6Al-4V, which is widely used in industry and transportation, is an example of such material. Rotary tool machining of titanium alloys has not been the subject of many studies. Additionally, if researchers were dissatisfied with their findings, the reason may not be the kinematic machining scheme itself but rather the tool design and the choice of cutting parameters. When tools are constructed of several components, inaccuracies in production and assembly can arise, resulting in deviations in the cutting part area. A monolithic driven rotary tool eliminates these factors. In the machining process, however, it may react differently from multi-component tools. The presented work focuses on the research of the technology for machining titanium alloy Ti-6Al-4V using a monolithic driven rotary tool. The primary goal is to gather data on the impact of cutting parameters on the machining process. The cutting force and the consequent integrity of the workpiece surface are used to monitor the process. The speed of workpiece rotation has the greatest impact on the process; as it increases, the cutting force increases, as do the values of the surface roughness. In the experiment, lower surface roughness values were attained by increasing the feed parameter and the depth of cut. This may predetermine the inclusion of a kinematic scheme in highly productive technologies.

## 1. Introduction

Conventional methods of machining materials with enhanced mechanical properties face many disadvantages, including a low cutting speed, short tool life, and time-consuming production [1,2,3]. To solve these issues, high-performance computing technology (HPC), high-speed machining (HSC), cryogenic cooling, and the use of specialized tooling systems are considered [4,5,6].

**Figure 1:**
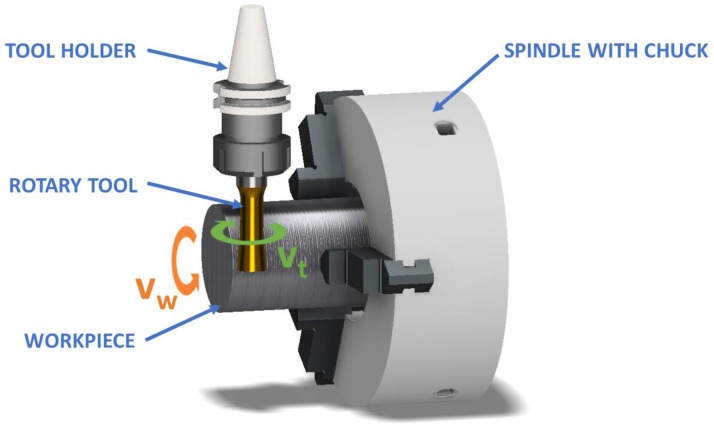
Scheme of actively driven rotation machining.

Rotary tool machining is mostly employed for the turning processes of materials with enhanced mechanical properties [7,8]. However, the cutting edge of conventional cutting tools wears down considerably in the machining process due to high temperatures, forces, and stresses [9,10,11]. It is feasible to prevent this problem by setting lower cutting parameter values. However, this results in an unwanted reduction in material removal, and an increase in the machining time and cost. Cutting fluids can reduce heat generation during machining, but due to the negative impacts on the environment and human health, attempts are currently being undertaken to include a more ecologically friendly alternative in the production process [12,13,14]. The usage of rotary cutting tools is one of these options (Figure 1). These tools differ in the kinematic machining scheme because they rotate the tool (*v_t_*) and the workpiece (*v_w_*) simultaneously [15]. Moreover, these rotary tools can be used in the milling process, where the rotary cutting insert is placed on the milling head [16,17].

When turning with a conventional tool, a high temperature is generated at the cutting point due to the fact that only one point of the tool is in constant contact, and wear is concentrated at this point as well. Rotary tool machining technology leads to increased material removal, reduced cutting tool wear [18,19], high cooling capacity [8,20], extended tool life [21], and better integrity of the machined surface. The rotary tool design might be monolithic or consist of a circular cutting insert clamped in a holder that rotates freely around its axis and serves as a continuous cutting edge along its entire circumference [22]. Each part of the cutting edge comes into contact with the workpiece for a short time during rotation and then cools in air due to rotational movement before again coming into contact with the workpiece. The heating and cooling phases alternate, which has a positive effect on the temperature and tool life while the wear is evenly distributed over the entire cutting edge. To prevent vibration during the turning process by the turning tool, the rigidity condition of the tool workpiece must be met [15].

In contrast to a self-propelled rotation tool, where the insert spins about its axis by chip formation, the actively driven rotation tool is driven by an external drive via a programmed spindle. While the self-propelled rotation tool can be used on any lathe, the actively driven rotation tool can only be used on a universal lathe with a milling spindle or on a multi-axis turning center. The tool with actively driven rotation can also be used in situations where a higher cutting speed and a larger removal of the chip cross-sectional area are required. When compared to a traditional turning tool, this type of tool has a longer tool life and is characterized by greater dynamic stability and rigidity throughout the cutting operation. Moreover, the application of a tool with actively driven rotation significantly reduces the production time; however, at the same time, it increases the wear due to the long continuous cuts during longitudinal turning. Nevertheless, the benefits of such a tool design exceed the disadvantages.

When compared to a conventional stationary tool, the use of a rotary tool is more cost-effective due to its ability to set a higher cutting speed, reduce tool wear, and reduce the manufacturing time.

Tools with self-propelled rotation are suitable for finishing operations, where the setting of a lower cutting speed and the removal of material with a small cross-sectional area are required [23]. Tools with self-propelled rotation become unstable and vibrate at higher cutting speeds and when removing material with a large cross-sectional area. Therefore, it is vital to adjust the cutting settings appropriately during the cutting process to ensure a longer tool life than a stationary turning tool would have.

Compared to a self-propelled rotation tool, an actively driven rotary tool provides significantly higher stability and rigidity in the cutting process. Increasing the penetration angles of the tool into the workpiece leads to an increase in the cross-sectional area of the chip to be removed [4].

### Machining of Titanium Alloys with Rotary Tool

Lei and Liu conducted an experiment to compare the high-speed machining of Ti-6Al-4V titanium alloy using a self-propelled rotation tool and a stationary turning tool [11]. Specifically, the tool life and the impact of the cutting insert rotation speed on the cutting force components and tool wear were investigated. For comparison, high-speed turning was performed with both tool types under the same cutting conditions and using the same cutting insert. The experiment proved that a tool with self-propelled rotation with sufficient rigidity and with a compact construction is suitable for machining titanium alloy at high cutting speeds. Compared to a stationary tool, the nominal tool life with a self-propelled rotation was approximately 37 times longer, and in the case of absolute tool life, it was more than 1.7 times longer. Olgun and Budak also conducted an experiment comparing stationary and rotary turning tools with respect to their durability, surface quality, and cutting force [24]. They found that better roughness in the feed direction was achieved with the rotating tool. Kossakowska and Jemielniak evaluated the machining of titanium alloy as unsatisfactory in their experiments. The use of a sufficient amount of coolant enhanced the machining results of Ti-6Al-4V to some extent. The best results were obtained for medium and high feeds, average cut depths, and high cutting speeds [25]. Based on this information, it is not possible to accurately determine suitable cutting parameters for the stability of the machining process. Another shortcoming in the previous works is the complexity of the construction of the tools. The task of the presented paper is to determine the influence of the cutting factors on the titanium alloy machining process and their importance.

## 2. Materials and Methods

In the current study, cutting tests were conducted to investigate the effect of the cutting parameters on the machining process with a driven monolithic rotary tool. The material of the workpiece was titanium alloy Ti6-Al4-4V.

### 2.1. Machined Material

Titanium is an important construction metal developed in the 1950s. Titanium alloys are widely used in a variety of fields due to their high strength, corrosion resistance, and heat resistance [26,27]. Although titanium is heavier than aluminum, it is also stronger in terms of specific weight. This predestines it for use mainly in aviation and other transport technology [28]. In numerous engineering applications, Ti-6Al-4V is one of the most extensively utilized titanium alloys with a guaranteed chemical composition (Table 1). It is a significant material for modern mechanical components and equipment, particularly in biomedical and aerospace systems, due to its outstanding corrosion resistance and high strength-to-weight ratio. However, machining of Ti-6Al-4V workpieces is complex and it is very difficult to produce components with the required shape and quality surface finish [29].

### 2.2. Monolithic Driven Rotary Tool

**Figure 2:**
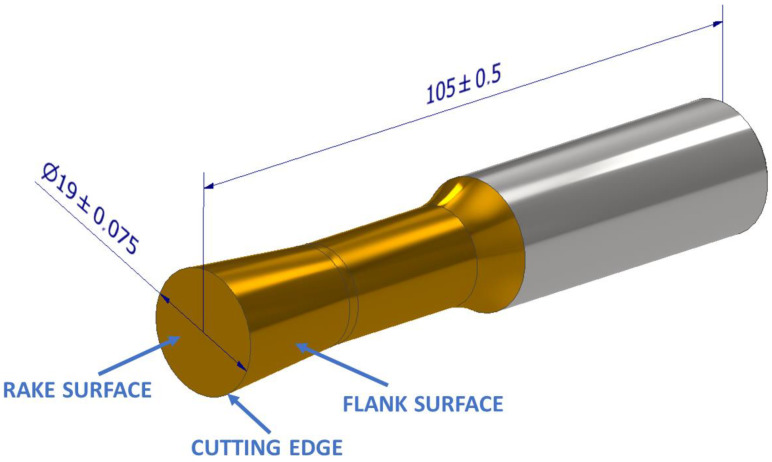
Model and basic dimensions (mm) of a monolithic rotary tool.

The monolithic driven rotary tool (Figure 2) was designed by the Department of Machining and Production Technology of the University of Žilina in Žilina. The geometry of the cutting part was modified in comparison with the proposed tool of the first generation [22]. The construction of the tool is monolithic and made of the material, sintered carbide. It consists of a cutting and clamping part. The cutting part of the tool is coated with TiN and consists of a circular cutting face and a conical back surface.

**Figure 3:**
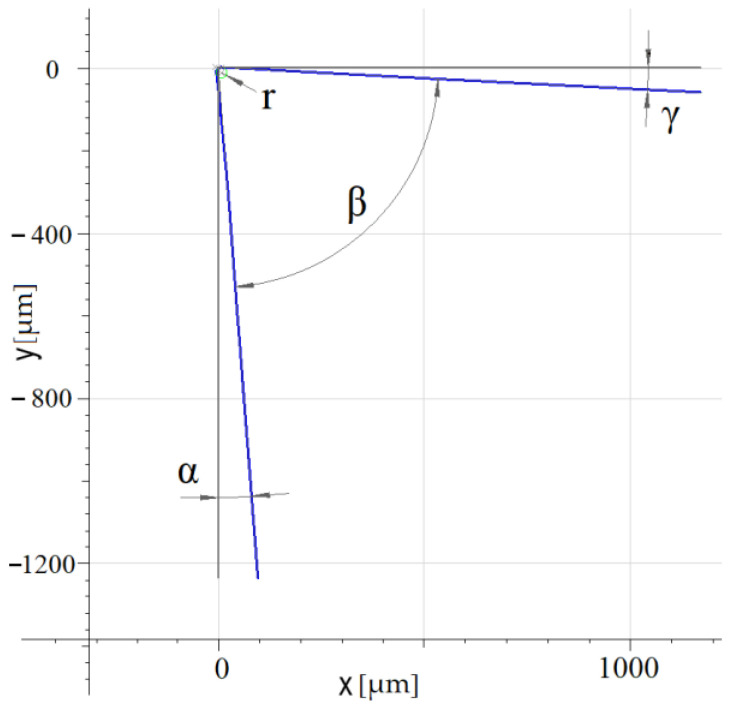
Recording wedge measurement of a monolithic tool.

The tool is clamped to the additional milling spindle and its speed and direction of rotation can be set via the NC program. The monolithic rotary tool is designed for CNC machines and turning of external cylindrical surfaces. Compared to the previous version of the tool, the cutting angles of the tool were adjusted in order to increase the rigidity of the cutting wedge. To verify the dimension precision of the tool production, the cutting part of the monolithic tool was measured using an Alicona Infinite Focus (Alicona Imaging GmbH Dr. Auner Straße 19 8074, Raaba, Graz, Austria), which is shown in Figure 3. Based on the performed measurement, the cutting angles of the tool were set at the angle of the rake surface *γ* = 3°, the angle of the flank surface *α* = 4.5°, and the angle of the cutting wedge *β* = 82.5°. The cutting edge radius was *r* = 13 µm.

### 2.3. Experiment Design

For turning with an active rotary tool, the same cutting parameters are required as for standard turning. The workpiece rotation speed (*v_w_*), feed (*f*), and cut depth (*a_p_*) are all included. Since this is turning with a rotary tool, the rotation speed of the tool must be defined (*v_t_*) and the tool rotation direction. The direction of rotation was used as shown in Figure 1. In this work, screening of the cutting parameters of machining was performed in order to determine the impact of their effect on the machining process. Therefore, the experiments were carried out only with a tool with a defined geometry (Figure 3) and no cutting fluid coolant. This eliminated the impact of tool geometry differences and the impact of cooling on the machining process itself. The levels of process parameters (*v_w_*, *v_t_*, *f*, and *a_p_*) were determined on the basis of the tool material, the characteristics of the kinematic scheme, the machinery used, and previous research work. The design of the experiment approach [30] was used to simplify the experiment, resulting in the 12 conditions shown in Table 2.

## 3. Experimental Part

**Figure 4:**
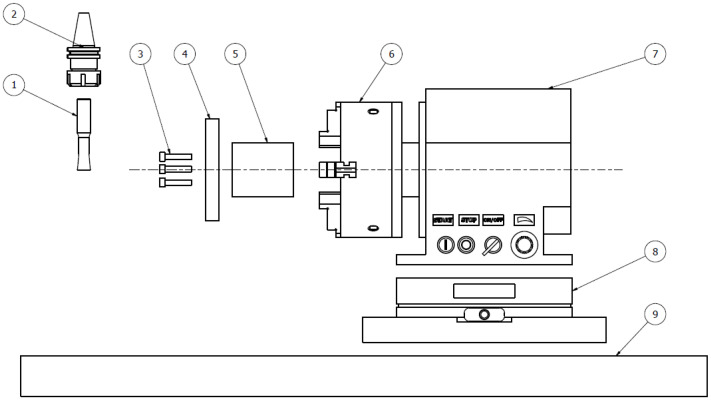
Setup scheme of the experimental environment.

In this study, circular cross-section samples of Ti-6Al-4V material with a diameter of 150 mm and a thickness of 20 mm were used, which are shown in Figure 4 under the position No. (4). This material was chosen since it is one of the most extensively used titanium alloys in the industry and has a wide range of applications. The sample had five holes, through which it was fixed to the clamping taper (5) by means of strength screws DIN 912 M8x40 (3). The clamping taper was placed by means of screw (6) in a horizontal machine spindle (7), which was mounted on a Kistler 9255A dynamometer (Kistler Eastern Europe Ltd., Prague, Czech Republic) (8). Dynamometer was on the workbench of the CNC machine (9). A tool holder (2) with a monolithic rotary tool (1) was placed in the vertical spindle of the machine. The cutting parameter levels and feed direction were controlled by the HURCO VMX30T CNC machine program (Hurco Company, Indianapolis, IN, USA).

**Figure 5:**
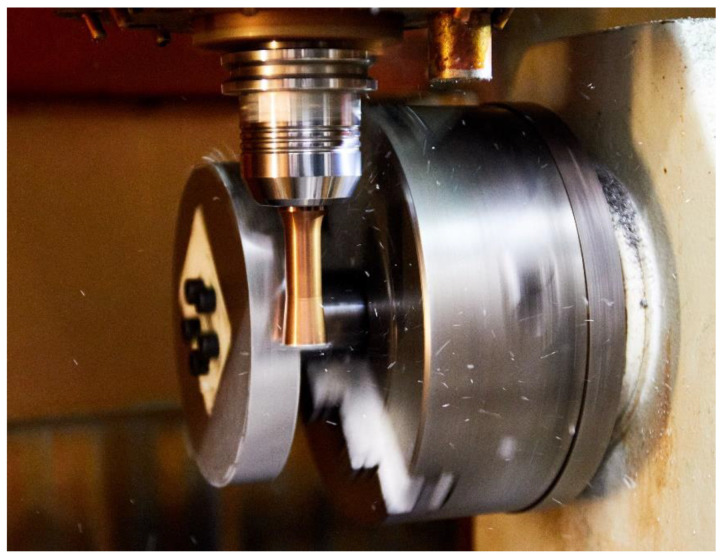
Machining process of Ti-6Al-4V with the rotary tool.

The measurement of the individual components of the cutting forces was performed using a KISTLER 9255A piezoelectric dynamometer. With respect to individual experiments, the resulting cutting force was defined by computation and its maximum value was calculated from the measured components of the cutting force (Fx, Fy, Fz). Before starting the turning process, the piezoelectric dynamometer had to be calibrated according to the manufacturer’s specifications. It was also necessary to determine the direction of action of the individual components of the cutting forces. The dynamometer continuously measured the components of the cutting forces even during the phase when the tool was not engaged. As a result, only those values were set aside for future processing when the tool was operating, and values were eliminated during the start-up and run-down phases. The design of the process parameters was used to set the parameters of individual experiments (Table 2). After performing 12 operations, the Alicona InfiniteFocus microscope was used to measure the machined surface. Figure 5 depicts the actual machining process using an actively driven rotation tool.

## 4. Results

The data from 12 experimental measurements of the machining process Ti-6Al-4V using a monolithic rotary tool was analyzed to obtain the reported results.

### 4.1. Cutting Force in the Machining Process

**Figure 6:**
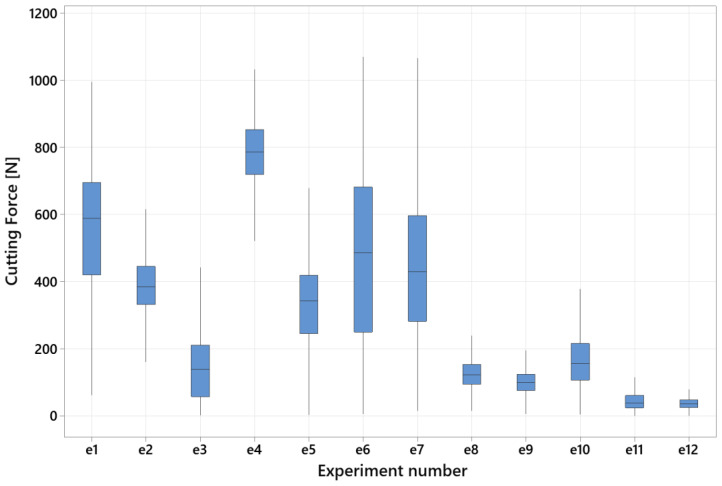
Total cutting force in individual experiments.

The measurement of the cutting force components and the subsequent determination of the total cutting force in the process allows monitoring of the force load. Based on this data, it is possible to monitor the impact of cutting parameters on the turning process with a rotary tool. It can be determined from the graph (Figure 6) that the largest range of total cutting force occurred during experiment No. 6. On the contrary, the smallest value of the total cutting force was measured during experiment No. 12 and its increase while turning was minimal. The maximum value in experiment No. 12 was 56.4 N. Setting of the cutting parameters to the lower limit resulted in a low cutting force value. The highest cutting force was measured during experiment No. 4, when the tool wear threshold was surpassed due to the high values of the cutting parameters and the low power of the externally driven spindle. The same situation occurred during experiment No. 7. During experiment No. 1, the value of the cutting force increased to 695.4 N and during experiment No. 4, to 852 N. The reason for the high cutting force was the setting of the cutting parameters at the upper limit.

**Figure 7:**
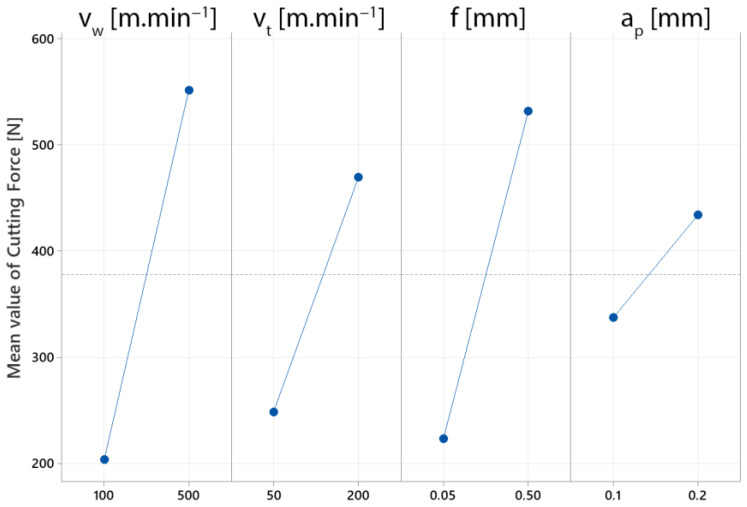
Main effects plot for maximal cutting force.

The main effects graphs (Figure 7) indicate the relative relevance of the parameters to the system response. If the line for a particular parameter is horizontal in the main impact graphs, the parameter had no significant influence. On the other hand, the parameter with the greatest inclination to the *x*-axis is the most pronounced. With a statistical accuracy of 85.90%, the graph of the main impacts provides interesting information on the impact of the cutting parameters on the final value of the cutting force in the machining process. It can be said that such accuracy is sufficient for screening the process parameters. We may conclude from the data that all the monitored parameters had an impact on the total value of the cutting force. The difference arises in the range of their impacts. The increase in the cutting force was least affected by the depth of cut (*a_p_*). This could also be due to the device’s low setting range. The workpiece rotation speed (*v_w_*) had the most significant impact on the strength of the cutting force. This phenomenon can also be assumed based on a theoretical point of view. In general, the workpiece’s rotational speed is a process parameter that has a significant impact on the cutting speed during machining. A significant difference in the cutting force values is also caused by the feed parameter (*f*). Based on the main impact data, we can state that an increase in the values of the process parameters resulted in an increase in the cutting force when turning with the rotary tool.

**Figure 8:**
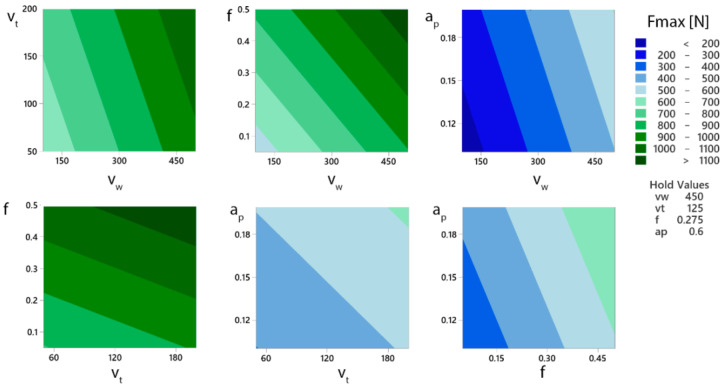
Contour (surface) plot of individual parameters for the total cutting force *Fmax*.

The contour plots (Figure 8) show the relationship between the two independent variables and the dependent variable, showing the values of the maximum cutting force *Fmax* for the combinations of the variables X and Y. The X and Y values are displayed along the X and Y axes while the contour lines and bands represent the *Fmax* value. The interaction of the parameters *a_p_* and *f* shows the smallest changes in *Fmax* values. This shows the *Fmax* bands with the widest gap. Thus, the change in the cutting force is attributable to *a_p_* and *f* in the range of 200 N at constant process parameters *v_w_* and *v_t_*. The most significant relationship is the combination of the workpiece rotation speed (*v_w_*) and feed (*f*) parameters. It is possible to determine these cutting parameters and achieve a reduction in the cutting force in the range of 500 N using the contour (surface) plot. The contour plot display shown here helps to determine the cutting parameters and their eventual optimization.

### 4.2. Quality of the Machined Surface

Each experiment was followed by measurement of the sample’s machined surface. The measurement was carried out using Alicona Infinite Focus equipment. This measuring device allows microscopic measurement of the surface roughness of the scanned surfaces. The measured sample was placed on the workbench of the device, aligned, and well lit. The measured section was located in the center of the sample. This eliminated the run-in and run-out surfaces after machining with a cutting tool. The device enabled us to create a surface scan, which was subsequently aligned, and the roughness parameter Rz was evaluated. Sampling took place on a defined area of 4 mm × 4 mm. The evaluation of the roughness parameters was based on five basic lengths with a size of 0.8 mm.

**Figure 9:**
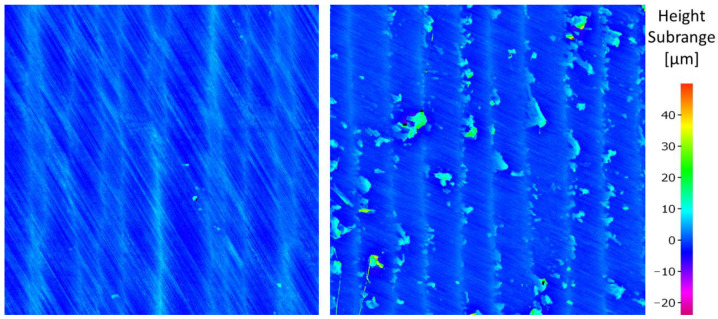
The final state of the machined surface of the Ti-6Al-4V material.

Figure 9 shows the comparison of the filtered surfaces of the samples from experiments 7 and 10, whose settings of the cutting parameters are given in Table 2. These are the samples for which the lowest and highest values of the roughness parameter Rz were achieved.

**Figure 10:**
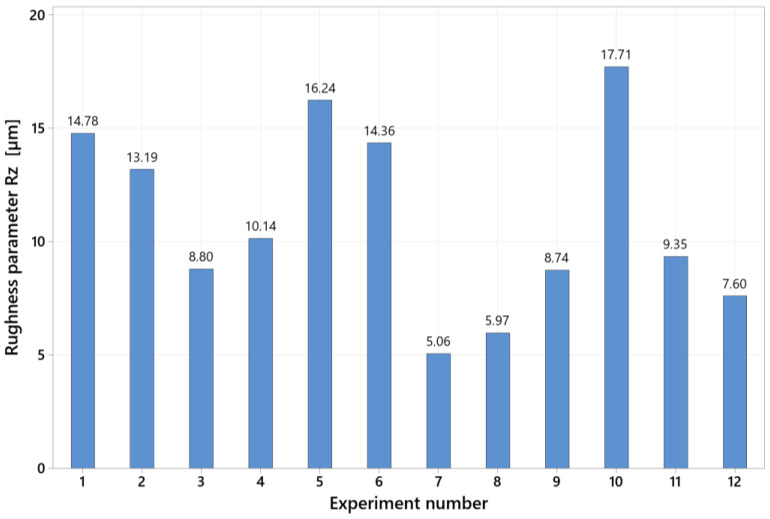
Values of the roughness parameter of the machined surface Rz.

Experiment No. 7’s process parameters, when combined with the rotary monolithic cutting tool, resulted in a stable machining process. There were no critical points on the machined surface and the machined surface was characterized by a regular texture. The chips were bonded to the machined surface in experiment No. 10. This results in the creation of protrusions on the surface and increases the values of the roughness parameter Rz. This phenomenon occurred mainly at the workpiece rotation speed (*v_w_*) set at the upper limit of the range and, at the same time, the low tool rotation speed (*v_t_*). The measured values of the roughness parameter Rz are graphically shown in Figure 10.

**Figure 11:**
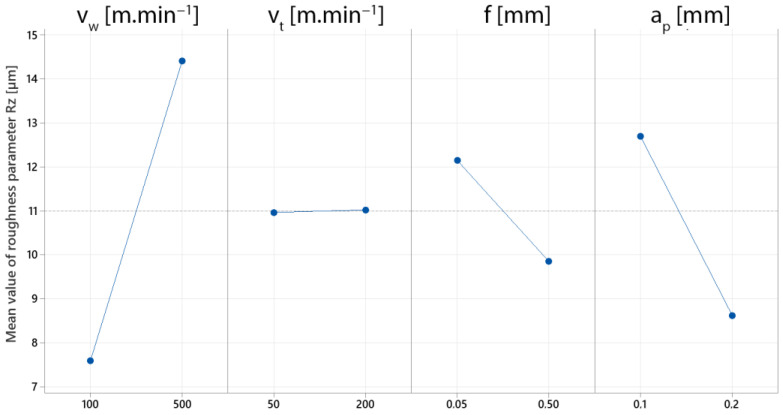
Main effects plot of the roughness parameter Rz.

During the screening, a graph of the main effects was created to better understand the influence of specific cutting parameters on the final surface roughness (Figure 11). This model has a statistical significance of 92.66 per cent. The most significant effect is the workpiece rotation speed (*v_w_*). Unevenness formed on the machined surface as this parameter was increased, resulting in higher values of the roughness parameter Rz. The almost horizontal line of the effect of the tool rotation speed (*v_t_*) indicates the insignificance of the given parameter for the resulting surface roughness. There are situations when increasing the depth of cut (*a_p_*) and feed (*f*) cutting parameters resulted in reduced surface roughness values. This phenomenon can be attributed to the kinematic scheme’s uniqueness, which is assumed to be a high feed machining technology.

**Figure 12:**
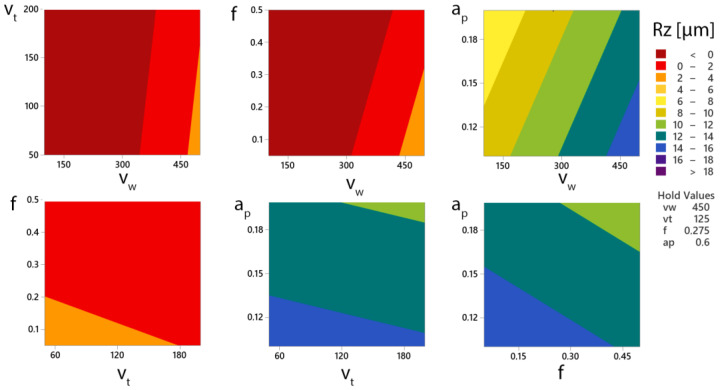
Contour (surface) plot of the parameter dependence on the roughness parameter Rz.

A contour plot of the surface roughness parameter Rz can be used to determine the cutting parameters to achieve the required surface roughness (Figure 12). A slight interaction occurs between the feed parameters (*f*) and the rotational speed of the tool (*v_t_*). The color map fields represent the range of Rz values. These have the highest range when the depth of cut parameter (*a_p_*) and the workpiece rotation speed (*v_w_*) are combined, with the surface roughness Rz changing by up to 10 µm depending on the parameter value.

The dependence of the selected cutting parameters and their influence on the roughness of the machined surface were determined by measuring and analyzing the experimental data. When the workpiece rotation speed was adjusted to the lower limit (*v_w_* = 100 m·min^−1^), lower Rz values were achieved. The quality of the machined surface was significantly influenced by the cutting parameters. As a result, it is necessary to understand how they interact and how to set them up properly.

## 5. Discussion

Turning of materials with enhanced mechanical properties, such as titanium alloy Ti-6Al-4V, remains a challenge that requires new technology to increase production and lower costs. The low cutting speeds and high wear of standard tools require an optimization process. Machining with an actively driven rotating tool is still not a common practice. Complex tool design solutions have high demands on production and assembly, which leads to the high cost of these rotary tools. The monolithic tool eliminates these shortcomings. Therefore, it is important to identify the technological possibilities of such a tool and examine its working process. To understand the technological possibilities of the given tool, it is necessary to set up the machining process itself. The presented work aimed to screen the cutting parameters in the process of machining titanium alloy, with an emphasis on the cutting force and the ensuing machined surface integrity.

The experimental studies and analyses showed that all selected cutting parameters (*v_w_*, *v_t_*, *a_p_*, *f*) have an effect on the overall cutting force during machining. The magnitudes of their effects, however, differ. The most significant effect occurred when the workpiece rotation speed parameter (*v_w_*) was modified. When this parameter was set at the upper limit, the highest cutting forces were also recorded. In experiment No. 4, these reached up to 851 N. The lowest cutting force was obtained when the process parameters were adjusted to the lower limit of the defined range, which varied from 25 to 51 N. The cutting parameters utilized in this experiment were several times higher than those employed in the work of Olgun and Budak [24], who specified a cutting speed of 45 m·min^−1^, a feed value of 0.1 mm, and a depth of cut of 0.2 mm, which was identical to the depth of cut used in our experiment. The machining of a titanium alloy with a rotary tool was determined to be inadequate by Kossakowska and Jemielniak [25]. The reason for this could be the design of the tool, the choice of an inappropriate geometry, or the process parameters.

Monitoring the machined surface revealed certain shortcomings in the experimental setup, as the chip adhered to the workpiece surface when the workpiece rotation speed (*v_w_*) was set. Of course, this phenomenon is an undesirable phenomenon that must be eliminated. Adjustment of the values of the cutting parameters or the application of process fluid can solve it. This phenomenon did not occur when the workpiece rotation speed was set to the lower limit, resulting in reduced surface roughness values Rz. The effect of the feed parameters (*f*) and depth of cut (*a_p_*) on the machined surface is a significant finding. In contrast to standard turning, where higher feed rates result in a degraded surface quality, lower Rz values have been achieved when machining with a new monolithic driven rotary tool. The depth of cut (*a_p_*) parameter reflected this effect as well. From this perspective, there was no significant effect of the tool rotation speed on the machined surface.

The non-standard kinematic machining scheme in combination with the new monolithic tool presents possibilities for advancing and increasing the productivity of machining titanium alloys. Based on the presented solution, it is possible to identify significant cutting parameters and their impact on the machining process by actively driven rotation.

## 6. Conclusions

The presented work focused on screening the influence of the change in the cutting parameters on the turning process of titanium alloy Ti-6Al-4V with an actively driven rotation tool. The kinematic scheme of turning with an actively driven rotation tool is different from the kinematic scheme of a conventional turning method. In addition to the main rotational movement of the workpiece (*v_w_*) itself, the tool rotates throughout the cutting process with the actively driven rotation rotary tool (*v_t_*). The tool itself, which was applied in the conducted experiments, was designed by the staff of the Department of Machining and Production Technology of the University of Žilina and is registered for patent recognition under the number: 3-2020. The used version of the tool was modified to meet the requirements for machining Ti-6Al-4V. The influence of the change in the cutting parameters was investigated in terms of the achieved cutting force and the size of the selected parameters of the roughness of the machined surface. The data were statistically analyzed and displayed graphically. From the experimental measurements and the gained data, we provide the following conclusions:

- The new monolithic rotary tool with a defined cutting wedge geometry is also suitable for machining materials with enhanced mechanical properties.
- Specialized CNC control is not required for real deployment (application) of the tool. However, the control system must allow for the control of at least three basic axes with the additional fourth axis (second spindle allowing independent rotation of the workpiece).
- With the appropriate setting of the process parameters, it is possible to use a kinematic scheme of actively driven rotation machining even without the presence of a cooling medium.
- The cutting parameters have a significant effect on the size of the cutting force; as their values increase, the cutting force also increases. The workpiece rotation speed (*v_w_*) has the most significant effect on the cutting force.
- With the appropriate settings, the dependencies of the cutting parameters and their interaction can result in a reduction in the total cutting force during the machining process.
- With a monolithic driven rotary tool, the state of the machined surface does not indicate critical values; hence, this kinematic machining scheme is suitable for standard turning tasks.
- When selecting cutting parameters in terms of the quality of the machined surface, it is possible to select higher values of the feed (*f*) and depth of cut (*a_p_*), which can lead to the process being included in high feed machining technologies.

Based on the experimental measurements and the reported results, we can conclude that the monolithic turning tool shows favorable results. Further research, possibly focusing on its critical load, temperature resistance, and durability, is required before it can be employed in industrial manufacturing.

## Figures and Tables

**Table 1:** Chemical composition Ti-6Al-4V based on attestation certificate (wt%).

Ti	Al	V	Fe	O	C
balance	5.50–6.75	3.5–4.5	0.40	0.20	max 0.08

**Table 2:** Process parameters and their values in the experiment.

StdOrder	*v_w_* [m·min^−1^]	*v_t_* [m·min^−1^]	*f* [mm]	*a_p_* [mm]
1	500	50	0.50	0.1
2	500	200	0.05	0.2
3	100	200	0.50	0.1
4	500	200	0.50	0.2
5	500	200	0.05	0.1
6	500	200	0.50	0.1
7	100	200	0.50	0.2
8	100	50	0.50	0.2
9	100	50	0.05	0.2
10	500	50	0.05	0.1
11	100	200	0.05	0.1
12	100	50	0.05	0.1

## Data Availability

The data that support the findings of this study are available from the corresponding author (R.J.), upon reasonable request.
